# Web-Based Study of Risk Factors for Pain Exacerbation in Osteoarthritis of the Knee (SPARK-Web): Design and Rationale

**DOI:** 10.2196/resprot.4406

**Published:** 2015-07-08

**Authors:** Joanna Makovey, Ben Metcalf, Yuqing Zhang, Jian Sheng Chen, Kim Bennell, Lyn March, David J Hunter

**Affiliations:** ^1^ Northern Clinical School, Kolling Institute, Institute of Bone and Joint Research, Department of Rheumatology, RNSH University of Sydney St Leonards, NSW Australia; ^2^ Centre for Health, Exercise & Sports Medicine Department of Physiotherapy University of Melbourne Melbourne Australia; ^3^ Clinical Epidemiology Research and Training Unit School of Medicine Boston University Boston, MA United States; ^4^ Northern Clinical School, Kolling Institute, Institute of Bone and Joint Research, Department of Rheumatology, RNSH University of Sydney St Leonards Australia

**Keywords:** knee osteoarthritis, Internet-based study, case-crossover design study, pain exacerbation, risk factors

## Abstract

**Background:**

Knee osteoarthritis (OA) is the most frequent cause of limited mobility and diminished quality of life. Pain is the main symptom that drives individuals with knee OA to seek medical care and a recognized antecedent to disability and eventually joint replacement. Many persons with symptomatic knee OA experience recurrent pain exacerbations. Knowledge and clarification of risk factors for pain exacerbation may allow those affected to minimize reoccurrence of these episodes.

**Objective:**

The aim of this study is to use a Web-based case-crossover design to identify risk factors for knee pain exacerbations in persons with symptomatic knee OA.

**Methods:**

Web-based case-crossover design is used to study persons with symptomatic knee OA. Participants with knee pain and radiographic knee OA will be recruited and followed for 90 days. Participants will complete an online questionnaire at the baseline and every 10 days thereafter (totaling up to 10 control-period questionnaires); participants will also be asked to report online when they experience an episode of increased knee pain. Pain exacerbation will be defined as an increase in knee pain severity of two points from baseline on a numeric rating scale (NRS 0-10). Physical activity, footwear, knee injury, medication use, climate, psychological factors, and their possible interactions will be assessed as potential triggers for pain exacerbation using conditional logistic regression models.

**Results:**

This project has been funded by the National Health and Medical Research Council (NHMRC). The enrollment for the study has started. So far, 343 participants have been enrolled. The study is expected to be finished in October 2015.

**Conclusions:**

This study will identify risk factors for pain exacerbations in knee OA. The identification and possible modification/elimination of such risk factors will help to prevent the reoccurrence of pain exacerbation episodes and therefore improve knee OA management.

## Introduction

Osteoarthritis (OA) is the most common joint disorder with more than 50% of people aged 65 years and older having radiological evidence of OA [1,2]. It is the leading cause of chronic disability in older adults with the risk of disability due to knee OA, the most commonly affected lower limb joint, greater than the disability due to any other medical condition [3,4]. It is anticipated that as the population ages and rates of obesity rise, the prevalence of OA will increase with some predictions suggesting that the number of people who suffer from OA will double by as early as 2020 [5].

Pain is the main symptom that drives individuals with knee OA to seek medical care and is a recognized antecedent to disability and eventually joint replacement [6-11]. The determinants of pain in OA are not well understood and are best considered in a complex framework of biopsychosocial factors [12,13]. The majority of persons with symptomatic knee OA experience recurrent pain exacerbations [14-16]. Factors or “triggers” that cause such exacerbations are not clearly identified, making it difficult to minimize reoccurrence of these episodes. A potential solution to managing this problem is to identify and control modifiable risk factors associated with knee OA pain exacerbations. Based on the aetiopathogenesis of knee OA [17], it is reasonable to speculate that factors that lead to either micro-structural joint damage or that decrease the pain threshold may exacerbate knee pain experienced by persons with knee OA.

Numerous studies have assessed the relationship of physical activity to the risk of developing radiographic knee OA with little or no attention paid to the relationship between physical activity and knee OA symptoms [18,19]. Different activities may pose varying degrees of risk for symptoms in knee OA. Some activities that are potentially of interest include prolonged standing [20,21], walking up and down stairs, and getting out of a chair. At present, there is a paucity of epidemiological data to explain which particular activities may contribute to increased pain severity.

Appropriate supportive footwear is recommended in treatment guidelines for the management of knee OA symptoms, although there is little trial evidence to support this [22]. Footwear can influence load through the lower limb [23-25] and potentially lead to pain in persons with knee OA. High-heeled shoes may be particularly problematic for women given that they increase compressive forces across the knee joint [26-29].

Among both genders, a history of injury to the stabilizing or load-bearing structures of the knee renders the joint highly vulnerable to radiographic OA in subsequent years [30]. Knee injury/trauma has been identified as the most important modifiable risk factor for knee OA in men, and is second only after obesity in women [31]; however, its relationship to increased pain in those with established OA has not been revealed.

Analgesic medication adherence is a known source of variation in pain control [32]. The absence of a cure and the chronicity of knee OA warrant continued adherence to prescribed therapy to maintain efficacy. The most widely used symptomatic agents for OA, the nonsteroidal anti-inflammatory drugs (NSAIDs) and COX-2 inhibitors, are associated with high rates of adverse events [33] and rarely relieve symptoms completely [34] which may contribute to poor long-term adherence. In turn, lack of consistent analgesic medication use may be associated with pain exacerbation.

Many believe that weather conditions can influence joint pain, but science offers little proof [35]. If the phenomenon were real, cause-and-effect mechanisms might provide clues aiding treatment of joint pain. Factors include ambient temperature, barometric pressure, relative humidity, sunshine, wind speed and precipitation; although the literature on the subject is sparse, conflicting, and vulnerable to bias [36]. While the biological mechanisms may not be fully understood, for patients who believe that weather can influence their pain, the effect seems to be real.

Pain is a highly subjective phenomenon, with a complex physiological and psychological basis [37]. A full understanding of pain requires consideration of psychological and social environmental processes mediating a patient’s response to their disease [38]. Helplessness, depression, stress, poor pain coping, self-efficacy, and the social context of arthritis are important considerations in understanding how people respond to their disease and pain management [39,40].

The Internet is a powerful platform that is increasingly being used in medical research [41,42]. Over the past few decades the Internet has had a major impact on research activities in various areas of health science [42-48]. The Internet can facilitate real time data capture at convenient times for participants without the practical limitations of traditional study methods. Online questionnaires have also been a useful recruitment tool for medical research projects through online social media network [47,49].

The scientific method best suited to identify a set of modifiable risk factors associated with knee OA pain exacerbations is the case-crossover design as it uses each case as its own control and is ideal for assessing the effects of triggers on recurrent episodic events [48,50-53].

We will therefore use a Web-based case-crossover design to evaluate a set of putative modifiable risk factors for pain exacerbation in people with symptomatic knee OA, including physical activity, footwear change, trauma and injury, medication use, climatic, and psychological factors.

## Methods

### Ethical Approval

Ethical approval has been obtained from the University of Sydney Human Ethics Committee (Protocol No: 14435), University of Melbourne Human Research Ethics Committee (HREC No. 0709220) and Radiation Safety Committee. All participants will provide either written or electronic informed consent.

### Design

This will be a Web-based case-crossover study. The case-crossover study [50,51] is a scientific method to answer the question, “What happened just before an event?”. In this study the event is a pain exacerbation in the context of symptomatic knee OA. The case-crossover design is analogous to a matched retrospective case-control design in which only matched pairs that are discordant for exposure contribute meaningful information. The information about possible triggers for pain exacerbation will be collected on a secure password-protected study website which will be located on a secure server. The website will display a consent form and administer the risk factor assessment questionnaires. Participants will be asked to complete these online questionnaires at the baseline and every 10 days for 90 days (control-periods) —a total of 10 questionnaires. They will be prompted to fill out these online assessments by means of automated reminder emails. The participants will also go to the website and complete pain exacerbation questionnaires when they experience an isolated incident of knee pain exacerbation (case-periods). Control-period questionnaires will obtain the frequency and levels of potential risk factors during the control-periods (no painful episode occurred).

Case-period (pain exacerbation) questionnaires will be completed by the participants when they experience what they believe to be a knee pain exacerbation during the three months of the study. Information about the frequency and levels of potential risk factors prior to the onset of knee pain exacerbation will be obtained. Risk factor assessment questionnaires will be the same for case-period and control-period online visits. The case-period questionnaires will only be available for those individuals who qualified as having a pain exacerbation based on the difference in knee pain level compared to the mildest intensity level reported previously at the baseline visit (≥2 point increase on the numeric rating scale [NRS]).

**Figure 1:**
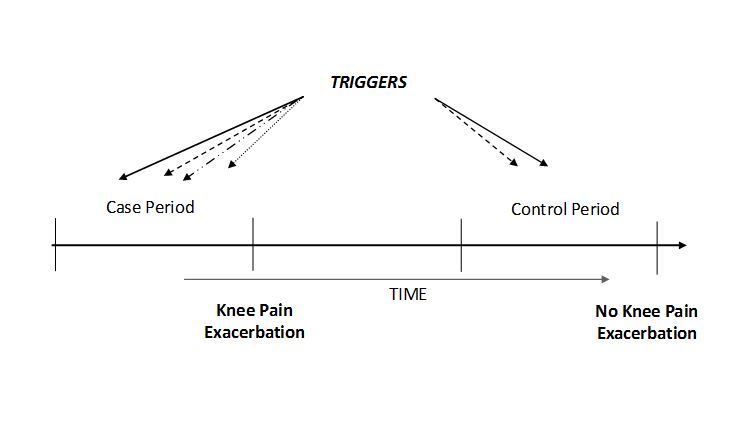
Case-crossover study design and timing of exposure measurements in relation to knee OA Pain Exacerbation.

The frequency/severity of each potential trigger/risk factor recorded during the case-periods will be compared with those that were reported during the control-periods within each participant (Figure 1).

### Participants

An online screening survey tool will be engaged for recruitment of eligible study participants. This tool will identify participants that qualify for the study based on their answers to eligibility questions. We will advertise the study on the official websites of Arthritis Australia, MyJointPain, Institute of Bone and Joint Research (University of Sydney), The Centre for Health, Exercise and Sports Medicine (University of Melbourne), and through Facebook.

We will email study information to the individuals from previous OA studies that have given their consent to be contacted for future research projects. We also will put the study advertisement in some Northern Sydney district local newspapers. When a potential study candidate registers his/her interest in participation through the screening survey tool, their contact details will be emailed to a study coordinator. The study coordinator will then contact the person for further assessment and enrollment if eligible. Prospective participants will also need to provide their most recent knee x-rays or their permission to access those at the imaging facility where they were taken. Once qualified based on the knee x-ray assessment by the study physician, the person will be enrolled and provided access to the study website.

Participants will need to provide their informed consent before accessing the study questionnaires. A choice of electronic or paper consent will be available. The electronic patient information page will appear on the study website when it is accessed for the first time. At the end of this website page participants will need to acknowledge that they have read and understood the study information and agree to participate by clicking on “I agree” button to proceed to study questionnaires.

To be eligible to enter the study, participants must be aged 40 years and over; have an active email address and access to a computer with the Internet; experience pain that fluctuates in intensity in at least one knee on most days in the past month; have x-ray evidence of knee OA (defined as presence of at least one area of definite tibiofemoral (Kellgren and Lawrence grade≥ 2) or patellofemoral osteoarthritis documented on a radiograph); have not had a knee joint replacement in the most painful knee or plan to have one in the next year and have never been diagnosed with rheumatoid arthritis or fibromyalgia.

### Risk Factors to be Assessed

#### Physical Activity

Physical activity (both recreational and occupational) will be assessed using the Seven-Day Physical Activity Recall (PAR) questionnaire; a standard, validated, and widely used measure of physical activity [54-57]. The PAR estimates an individual’s time spent on physical activity, strength, and flexibility activities for the last seven days. Physical activity will be grouped into three categories: (1)“moderate” intensity activities that produce feelings similar to those accompanying brisk or fast walking; (2) “very hard” activities that produce feelings similar to those of running or jogging; and (3) “hard” activities that produce feelings that are between the feelings that go with moderate and very hard activities.

#### Footwear

Images of shoes/inserts, including shoes with heels > 5cm will be posted on the study website. Participants will report which type(s) of shoes they have worn during the last 48 hours and how long they were worn for. This questionnaire was used successfully in the large Framingham Foot Study [58].

#### Trauma and Injury

We will ask questions on whether any fall, injury, buckling or trauma occurred in or around the knee during the preceding 48 hours. These self-reported instruments have been widely used and validated in prior studies of buckling [59], injury/trauma [60], and falls [61].

#### Medication Use

Participants will report medication use for each of the case and control-periods by choosing medication names from the list provided in the study questionnaires. The list includes oral, topical, and injection medications for pain (including nonsteroidal anti-inflammatory drug (NSAID), opioids, other analgesics, and steroid and hyaluronic acid injections), and medications taken for other medical conditions that previously have been reported as related to OA pain. We will ask questions about the dose and frequency of these medications for yesterday, the day before yesterday, and 3-7 days ago. The information about the use of natural remedies and complementary therapies will also be collected based on the same time frame.

#### Weather

Specific climatic data (ie, barometric pressure, ambient temperature, and humidity)will be downloaded from the Australian Bureau of Meteorology website for the geographical location provided by the participant. We will ask participants about their location in each of the last two days. We will collect information about the time they spent in the air-conditioned environment and outside. The information about participant’s travel destinations and time spent on a commercial jet in the last two days will also be recorded.

#### Psychological Factors

We will assess the following psychological factors using validated questionnaires: positive and negative effect/mood and catastrophizing every 10 days, and coping strategies, coping efficacy, perceived stress, and social support every 30 days. Participants will complete the Positive and Negative Affect Schedule (PANAS) Questionnaire [62] for positive and negative effect/mood measurements. Pain catastrophizing will be measured using the pain catastrophizing scale [63]. To assess the use of coping strategies, participants will complete Stone and Neale's Daily Coping Inventory (DCI) [64-66] adapted for chronic pain coping. Coping efficacy will be assessed on a scale from the coping strategies questionnaire [67,68]. The perceived stress scale will be used to measure the degree to which situations in participant’s life are appraised as stressful [69]. We will assess the amount of support that participants receive from their friends and family using the Lubben social support questionnaires [70,71].

Study questionnaires for the baseline, the control-period, and case-period are shown in Table 1.

**Table 1:** Study website questionnaires.

Questionnaires	Baseline	Control-period	Case-period
Demographics	x		
Comorbidities	x		
Perceived risk factors	x		
Baseline pain characterization -Index knee	x		
Baseline pain characterization -Contralateral knee	x		
Medications	x	x	x
Lubben social support	x	x	x
Knee injury and Osteoarthritis Outcome Score (KOOS) - Index knee	x	x	x
Knee Injury and Osteoarthritis Outcome Score (KOOS) - Contralateral knee	x	x	x
Intermittent and Constant Osteoarthritis Pain (ICOAP)	x	x	x
Physical activity	x	x	x
Footwear change and heel height	x	x	x
Trauma/injury/knee buckling	x	x	x
Climate	x	x	x
Pain Coping Inventory (PCI)	x	x	x
Perceived Stress Scale (PSS-10)	x	x	x
Daily mood measured by using Positive and Negative Affect Scale (PANAS)	x	x	x
Pain flare			x

### Pain Outcome Measures and Exacerbation Definition

The primary outcome measure is the level of knee pain. We will assess pain level using the numeric rating scale (NRS) for pain, which is a commonly used, valid, and reliable measure [72-74]. The pain NRS is a single 11-point numeric scale ranging from 0 - “no pain” to 10 - “the worst pain possible” [72,73]. At the baseline we will ask the participants to indicate how severe their knee pain is at its mildest, usual, and worst times of their current everyday life. A pain exacerbation will be operationally defined as occurring if the pain measure is 2 points higher than it was at its mildest intensity reported at the baseline visit. Participants are instructed to provide the information about every episode when they have a disabling increase in their knee symptoms that lasts for longer than 8 hours without settling. When participants log onto the study website to report these episodes they will be asked what level of pain they are experiencing “right now”. The online questionnaire will automatically determine if the current episode is a pain exacerbation based on the difference in pain level on the NRS from the previously collected NRS data. Participants will be given instruction to report any episode when they have a disabling increase in their knee symptoms that lasts for longer than 8 hours without settling. To avoid subjectivity they will not be aware of how the pain exacerbation is evaluated.

### Sample Size Calculations

Study sample size was calculated based on case-crossover study design. A sample size of 146 participants will have 80% power at 95% confidence level to detect an OR of 2 for knee pain exacerbation in the case-period relative to control-period if the probability of exposure (trigger/risk factor) among control-periods is at least 0.1 and the correlation coefficient for the exposure between matched case-periods and control-periods is not more than 0.3. We will recruit over 300 participants allowing for approximately 30% of participants who may not experience a pain exacerbation due to natural course of the disease or early withdraw from the study. The sample size estimation is conservative because we assume that each participant only provides exposure information for one case and one control-period whereas in reality participants will provide data from multiple case-periods and multiple control-periods. Given the different risk factors being measured, we will not combine these in the analysis and will treat them as independent predictors.

### Statistical Analysis

We will assess the relation of risk factors to the risk of knee pain exacerbation by conditional logistic regression analysis for matching model (m:n matching —as each selected participant could have multiple case and multiple control-periods) by using SAS software version 9.4. Only participants with both case and control-periods will be included in the regression analysis. Descriptive statistics such as total number, mean (standard deviation), median (range or interquartiles) or proportions will be used to summarize the data. For categorical exposure variables we will keep the original categories. For continuous exposure variables we will classify responses into categories based on predetermined cut-points. We will initially use narrow exposure categories to identify patterns of association between the risk factor and pain exacerbation; although it may be necessary later to collapse some of these categories to obtain stable estimates. Odds ratios (OR) and 95% confidence intervals (CI) for the risk factors will be reported using the Mantel-Haenszel method [75]. We will also evaluate the joint effects of several risk factors on pain exacerbation by either comparing OR in subgroups, defined by different levels of the potential effect modifier or using multiple conditional logistic regression models. Subgroups will be compared with the chi-square test for homogeneity, or test for interaction of various risk factors.

While few data are available on the likely effect-period for each risk factor for pain exacerbation, the actual duration of the effect-period can be inferred empirically by examining the change in magnitude of the OR under different assumptions about duration. Thus, we will calculate the OR by assuming the effect-time period to be one day and then two days. The better estimate of duration is the one with minimal nondifferential misclassification (ie, one that maximizes the OR estimates) [50,52]. Self-matching of cases eliminates the threat of control-selection bias and increases efficiency.

As with any study conducted on human participants those with missing values would not contribute, or contribute less data to the effect estimates. We will take various approaches to minimize loss to follow-up, including comparison of the characteristics of those who provide the complete follow-up data with those who only provided part of the data, performing stratified analysis among those who have completed data points and among those who do not have complete data points, to see if the effect estimates vary.

## Results

This project has been funded by the National Health and Medical Research Council (NHMRC). The enrollment for the study has started. So far, 343 participants have been enrolled. The study is expected to be finished in October 2015.

The data obtained during the course of the study will be presented in separate manuscripts for each of the studied knee OA pain exacerbation risk factors and their possible interactions.

## Discussion

This study will use an Internet-based case-crossover design to identify potential risk factors or “triggers” for pain exacerbation episodes in people with knee OA, including physical activity, footwear change, trauma and injury, medication use, climatic, and psychological factors. This study design is best suited to answer these questions as it uses each case as its control and is ideal for assessing effects of triggers on recurrent episodic events, such as knee OA pain exacerbations [51].

The completion of this study will identify risk factors for pain exacerbations in knee OA. The identification and possible modification/elimination of such risk factors will help to prevent the reoccurrence of pain exacerbation episodes in the future and therefore improve knee OA management.

Possible limitations of the study include incomplete data in questionnaires, in addition to some potential for recall bias and participant fatigue. Every participant will be followed regularly for 90 days and it is possible that they may not report every pain exacerbation that they experience during that time. Another possible limitation is that the study cohort will include Internet-users only and the results may not be generalizable to all people with OA.

A notable strength of this study includes the real time capture of data prior to pain exacerbation and an Internet-based case-crossover study design.

The findings from this study will contribute to better understanding of the pathophysiology of pain exacerbation in knee OA, and guide the development of rational management strategies to prevent its occurrence. Identifying modifiable risk factors for pain and avoiding these factors could improve the quality of life for millions of people with knee OA and have great public health importance. If practitioners are armed with information about appropriate shoe wear, adverse physical activities, regular medication compliance amongst other factors that will be assessed in our study, this information will be important to counsel patients about during typical clinical encounters. It could also be used to direct self-management strategies and target appropriate treatments including psychological interventions. Thus, this work has immediate clinical applicability.

## Abbreviations

CI: confidence intervals
ICOAP: Intermittent and Constant Osteoarthritis
KOOS: Knee injury and Osteoarthritis Outcome Score
NHMRC: National Health and Medical Research Council
NRS: numeric rating scale
NSAIDs: nonsteroidal anti-inflammatory drugs
OA: osteoarthritis
PainPCI: Pain Coping Inventory
PSS: Perceived Stress Scale
PANAS: Positive and Negative Affect Scale
OR: odds ratios

## Appendix

Multimedia Appendix 1 NHMRC Peer-review report.
