# Supplementary material for: Web-Based Study of Risk Factors for Pain Exacerbation in Osteoarthritis of the Knee (SPARK-Web): Design and Rationale
Source: JMIR Res Protoc. 2015 Jul 8;4(3):e80. doi: 10.2196/resprot.4406 (PMC4526980; doi:10.2196/resprot.4406)
Supplement: Multimedia Appendix 1 [file resprot_v4i3e80_app1.pdf]

Australian Government
National Health and Medical Research Council
NHMRC
WORKING TO BUILD A HEALTHY AUSTRALIA
www.nhmrc.gov.au

Professor David Hunter
University of Sydney
Rheumatology Department
Level 4, Block 4
Royal North Shore Hospital
St Leonards NSW Australia 2066

Dear Professor Hunter

NHMRC Project – APP1021655 – Web based study of risk factors for pain exacerbation in knee osteoarthritis.

Thank you for applying for a NHMRC Project for funding in the year 2012. Please find attached the assessments for your application (identified above). You are now invited to respond to those assessments.

As in previous years, NHMRC sought two external assessments for your application. We received two such assessments for most applications and more than two for a relatively small proportion. In some instances, however, our requests for external assessments were declined and you may find that there are fewer than two.

We have provided all written assessments received for your application. Your responses to the comments in these assessors' reports will be provided to GRP members.

As your application will proceed to review by a Grant Review Panel (GRP), your Applicant Response must be **received by NHMRC within 7 working days** of the day after the date on your Assessment Rebuttal Letter. This will ensure that your response can be considered by the GRP.

No Application Response will be accepted if lodged after the 7 working day time limit. If this timeframe raises significant issues, or if you experience other problems with submitting your Response, please contact NHMRC's Research Help Centre (help@nhmrc.gov.au; Tel. 1800 500 983). Information on how to submit your Response is provided below.

To assist in the further consideration of your Response, it is important that you follow the requirements set out below, including in relation to the submission of your Response (see instructions at end of this letter). If you do not adhere to these requirements, the Response may not be considered by the GRP:

**Format:**	A single document converted into a PDF file that **<u>must not</u>** exceed **2Mb** in size. Applicants and RAOs are advised to retain a copy of the response, including a copy of the PDF file they submit**.**
**Page Limit:**	Not more than 2 pages.<br><br>If the application is going to be assessed by the Indigenous Health Research Grant Review Panel (IGRP), applicants will be advised in a separate message. If this is the case, applicants will be permitted to use **an additional third page.** This additional page enables applicants to respond to *The Criteria for Health and Medical Research of Indigenous Australians* (refer: http://www.nhmrc.gov.au/_files_nhmrc/file/grants/indighth.pdf).<br><br>References and updates to the Chief Investigator's publication list must be included within the page limit.
**Paper Size:**	Standard A4 (210 x 297mm).
**Title:**	Response should be titled "Applicant Response" in centred font with the Application ID in the top right hand corner.
**Margins:**	All margins must be <u>at least</u> 2.0cm.
**Font:**	At least 12 point and Times New Roman only.
**Line spacing:**	Must be set to single or greater.
**Character spacing:**	Spacing must be set to normal. Scale must be set to 100%.
**Web Links:**	Do not include links to additional information on any website in the Applicant Response, excluding references to published peer review journal articles that are only available online.
**Graphics:**	Graphics (pictures, diagrams etc) may be included in the response. The Applicant Response may be printed and reproduced in black and white and any colour graphics must be visible when reproduced in black and white.
**Tables:**	Tabulated information containing text is not considered to be an image or diagram. Text within tables must comply with the above requirements concerning fonts and spacing.

**Labelling Graphs and Images:**	Axes of graphs and labels of parts of images may be in a reduced font. However, the description and/or legends of all graphs and images must comply with the above formatting requirements.
**File name:**	The PDF file must be named in the following format:“ApplicationID - CIA Surname - Applicant Response.pdf” (for example: APP9011023 - Smith - Applicant Response.pdf).

We encourage use of the “Applicant Response Template for Project Grants” available at http://www.nhmrc.gov.au/grants/apply/projects/index.htm#a6 and in the RGMS library, when preparing your response. Note that this template:

a) has very restrictive text formatting. For example the use of bulleted lists, bold, italicised or underlined text within a paragraph is not possible.
b) must be converted to a PDF file and must comply with all other requirements detailed above, before uploading into RGMS.

Please ensure your response is a single document of not more than two pages (three pages for applications being assessed by the IGRP) that has been converted into a PDF file not exceeding 2Mb in size.

How do I submit my Applicant Response (rebuttal)?

1. Log into RGMS (www.rgms.nhmrc.gov.au)
2. From the left hand menu select ‘*Results and Rebuttals*’
3. Locate your application using the relevant filter fields.
4. Click on the properties icon for the application you wish to view.
5. Select the ‘*Rebuttal*’ tab
6. In the left hand sub-menu click on ‘*Rebuttal*’
7. Use the ‘*Browse*’ button to locate your rebuttal file. Click ‘*Save*’ to upload the file to RGMS.
8. In the left hand sub-menu click on ‘*Submit my Response*’
9. From the drop-down list select ‘*Yes, Submit my response to NHMRC*’
10. Click ‘*Save*’
11. Your rebuttal has now been uploaded in RGMS and submitted to NHMRC.

Yours sincerely

[Authorised for electronic distribution by:]

Tony Willis
Director, Project Grants
Research Programs

25/07/2011

Australian Government
National Health and Medical Research Council
NHMRC
WORKING TO BUILD A HEALTHY AUSTRALIA
www.nhmrc.gov.au

PROJECT SCHEME 2011
-
ASSESSMENT REPORT

APPLICATION DETAILS

**Application ID**	APP1021655
**CIA Name**	Professor David Hunter
**Application Title**	Web based study of risk factors for pain exacerbation in knee osteoarthritis

Assessor 1
01. Scientific Quality (This includes the clarity of the hypothesis or research objectives, and the strengths and weaknesses of the research plan and the experimental design. It also relates to the feasibility of the proposed research; that is, is it able to address successfully the stated hypothesis and objectives. For further detail please refer to section 10.1 of the Project Grants Funding policy for funding commencing in 2012.)
The design is innovative and appropriate to answer the study aims. I have a small number of questions I would like you to respond to:

You are enrolling a convenience sample of past participants from your research studies who have access to the internet. To what extent can you generalise your results to Australians with knee OA?

In a simple case-crossover design there is one case and one control window but your design is more complex. How does the statistical analysis plan deal with four planned control observations (but perhaps less if subjects are non-compliant) and an unknown number of case windows in the 6 month study period?

You are using the baseline pain score to define the usual level of pain and this score is then used to define a subsequent exacerbation. The application is silent on the possibility that a participant may be experiencing an exacerbation at baseline. How will you deal with this issue? How will you deal with the possibility of an exacerbation coinciding with the 2, 4 and 6 month control window assessments?

You have clearly defined the onset of an exacerbation episode but not the resolution. What is this definition?

Assessor 1

02. Significance and Innovation (This includes the potential to increase knowledge about human health, disease diagnoses, or biology of agents that affect human health, or the application of new ideas, procedures, technologies, programs or health policy settings to important topics that will impact on human health. For further detail please refer to section 10.1 of the Project Grants Funding policy for funding commencing in 2012.)
This is a novel and important study. Identifying factors that trigger an exacerbation of knee OA would be a breakthrough that would help a substantial number of people worldwide.

Assessor 1
03. Track Record - relative to opportunity (Track record is considered in relation to opportunity – with regard to factors such as career disruptions, administrative and clinical/teaching load, and typical performance (including publications) for the field in question. For further detail please refer to section 10.1 of the Project Grants Funding policy for funding commencing in 2012.)
The three CIs have outstanding international track records in the rheumatology/musculoskeletal field. AI Bennell and Zhang also have outstanding track records and are well suited to the study with clear roles.

Assessor 1
Budget comments
The budget seems reasonable except for asking for computers. This is an indirect cost item according to the Project Grant Funding Policy (page 43).

Assessor 2
01. Scientific Quality (This includes the clarity of the hypothesis or research objectives, and the strengths and weaknesses of the research plan and the experimental design. It also relates to the feasibility of the proposed research; that is, is it able to address successfully the stated hypothesis and objectives. For further detail please refer to section 10.1 of the Project Grants Funding policy for funding commencing in 2012.)
Disease definition:Poor. Which of the 3 ACR criteria for knee OA is being used? Is the method of diagnosis(ie from Australian GP notes) validated?
Definition of exacerbation:Evidence for its validity? What % of patients self report a range of pain of >100units on WOMAC? What is the evidence that pain changes to this extent spontaneously, as it is similar to the effect size seen in NSAID trials? Is there data regarding the frequency of exacerbations used in power calculations?
Risk factors:Background does not accurately reflect what is already known about many of these, thus how novel will the results be? Esp given the existing data (including Australian data) available.
Limitations of prior studies persist in the application eg relating to weather
Is the study powered to examine trauma in the 6 months, given its frequency? Is the tool appropriate for this purpose?
Do the dimensions to be measured using the psychological tools change over 2 months, esp without an intervention? Whilst these tools are valid, are they validated to be used this way?
Provided references regarding validity of risk factor measurement are inaccurate eg ref 50 does not mention footwear

Recruitment:Are database subjects involved in other studies as this precludes involvement? If subjects do not report a range of pain of >100 at baseline, how likely are they to have an exacerbation as defined?
Compliance:on what basis is it assumed that participants will log on for every exacerbation? What, if compliance is <100%, is the effect on power?
A Hx:method not tested for OA exacerbation, validity of measure
B Plan:appropriate study design for question, deficiencies in measures and recruitment are major
C Feasibility:significant concerns, 1)diagnosis of OA is not validated, 2)what is the justification for the use of the effect size upon which the study has been powered? 3)subject learning, given repeated measures? 4)statistical methods:what is the power to detect interactions as is planned?

Assessor 2
02. Signifigance and Innovation (This includes the potential to increase knowledge about human health, disease diagnoses, or biology of agents that affect human health, or the application of new ideas, procedures, technologies, programs or health policy settings to important topics that will impact on human health. For further detail please refer to section 10.1 of the Project Grants Funding policy for funding commencing in 2012.)
This is an important area, with significant clinical utility. Whilst the answers to the proposed questions would have impact, there are a number of methodological issues that are of concern. The risk factors to be examined are not novel, and if measured as described, are not appropriate to answer the question as it is posed. Are the measurement tools (esp psychological tests) designed to be used as frequently as proposed? What is the evidence that learning does not occur? What is the evidence that these psychological measures (eg factors change over the short term, particularly without an intervention? There are gaps in the background.
Whilst the study design (Case crossover) is appropriate to answer the question, and if well performed, would add to the literature however, as neither the definition of disease nor exacerbation are validated, the potential impact of is diminished. This differs considerably to the investigators previous study of gout. On what basis do the authors base 100% compliance with logging on at the time of an exacerbation? How does compliance for subject initiated measures differ to regular review?

Assessor 2
03. Track Record - relative to oppurtunity (Track record is considered in relation to opportunity – with regard to factors such as career disruptions, administrative and clinical/teaching load, and typical performance (including publications) for the field in question.For further detail please refer to section 10.1 of the Project Grants Funding policy for funding commencing in 2012.)
CIA has an excellent track record, with respect to publication and grants, largely based on work performed overseas. The 29 conference abstracts which are currently included as accepted for publication should be removed.
CIB and CIC both have excellent track records, with national and international standing.

Assessor 2
Budget comments

The budget is generous, with PSPs requested at higher levels than would be necessary to recruit, and manage study. The xrays are costed at above the rebate. It is not justified that radiologists are required to read xrays again: the senior (or junior) PSPs would be able to be trained to score xrays – additional radiologists' payments are not required.
Financial reimbursement for completion of questionnaires is not usual.
Are there Australian Programmers who could do the same job as New England Survey Systems?

Assessor 2

Overall comments

Is there data to validate the main outcome of the study?
Is there data to justify the frequency of exacerbations, as this is required to perform a realistic sample size calculation?
Have the measures of the risk factors to be tested been validated for frequent testing? Have the risk factors been shown to change over short periods of time, without an intervention, as is proposed in this study? Can these small changes be captured by the methods proposed? For the psychological tests, is there evidence that repeated questionnaire completion does not affect the way a participant completes questionnaires? For questions regarding injury, how frequently are these injuries likely to occur to a population such as the proposed study population? If these are infrequent, how likely is this study to be able to answer this question?
Also see questions in previous sections.
